# Measurement of Fetal Abdominal and Subscapular Subcutaneous Tissue Thickness during Pregnancy to Predict Macrosomia: A Pilot Study

**DOI:** 10.1371/journal.pone.0093077

**Published:** 2014-03-27

**Authors:** Ling Chen, Jing-Jing Wu, Xiao-Hui Chen, Li Cao, Yun Wu, Li-Jun Zhu, Kang-Tai Lv, Chen-Bo Ji, Xi-Rong Guo

**Affiliations:** 1 Nanjing Maternal and Child Health Hospital of Nanjing Medical University, Nanjing, China; 2 Institute of Pediatrics, Nanjing Medical University, Nanjing, China; Michigan State University, United States of America

## Abstract

This study assessed the growth trends and reference ranges of the ultrasound parameters, fetal abdominal subcutaneous tissue thickness (ASTT) and subscapular subcutaneous tissue thickness (SSTT), in the last two trimesters of normal pregnancy in a Chinese population. We recruited 744 healthy women with singleton pregnancies. The ASTT and SSTT were evaluated at different times between 21 and 36 weeks of gestation. The correlations between these parameters and fetal gestational weeks were assessed using linear regression analysis. Both ASTT and SSTT increased with gestation, and both parameters showed a strong correlation with gestation (ASTT vs. GA, R^2^ = 0.792; P<0.0001; SSTT vs. GA, R^2^ = 0.302; P<0.0001). Time-specific reference ranges, including 5^th^, 50^th^ and 95^th^ percentiles and means ± SD, were constructed for ASTT and SSTT. These results provide a preliminary reference range to evaluate whether fetal development and maternal metabolic health is normal or not in a Chinese population.

## Introduction

For many aspects of perinatology, it is important to ensure that the fetal growth rate is appropriate for the age of the fetus. Abnormal fetal growth can cause prenatal and postnatal complications, and is associated with increased neonatal morbidity and mortality [1], [2]. Therefore, evaluation of fetal intrauterine growth by ultrasound measurements is advisable.

A range of ultrasound anthropometric parameters are used to determine normal fetal growth [3], [4]. Of these, fetal abdominal circumference or fetal weight calculated using a combination of ultrasound-derived parameters, is commonly used [5], [6]. However, both of these measures have a limited sensitivity and specificity. As Cetin et al. reported, the percentage error of these methods of fetal weight estimation could be as high as 25% [7], due to technical measurement errors and erroneous assumptions of fetal density [3], [4]. Additionally, the fetal abdominal circumference measurement only predicts 78% of macrosomic fetuses [8]. Therefore, researchers have been investigating the usefulness of another sonographic measurement, subcutaneous tissue thickness (SCTT), taken at a range of locations on the fetal anatomy [3], [9]–[11].

Recently, studies have shown that SCTT measurements, either on their own or incorporated into conventional fetal weight prediction formulae, could be used to evaluate fetal growth, and, in addition, assess whether maternal glucose levels are normal. In a comparison of SCTT between fetuses from a group of mothers with gestational diabetes and those from a normal control group [10], [11], there were significant differences between initial fetal SCTT, but no difference after the mothers had been treated for diabetes. The measurement of fetal SCTT gives a more accurate estimation of the stability of maternal glucose levels than a maternal ambulatory glycemic profile [12], [13]. Meanwhile, Buhling et al. found a direct correlation between intrauterine sonographically determined subcutaneous tissue thickness and postnatal caliper skinfold measurements [14], indicating that measurement of intrauterine fetal SCTT is a reliable method to determine the thickness of fetal subcutaneous fat.

The aim of this study was to assess the growth trends and reference ranges for fetal SCTT during gestation in a Chinese population, to provide information about their use as predictors for fetal macrosomia and maternal gestational diabetes. As the thickness of fetal subcutaneous fat tissue at the abdomen (ASTT) and scapula (SSTT) are the best predictors of fetal macrosomia [15], these measurements were obtained by ultrasound between 21 and 36 weeks of gestation in a Chinese population of normal, healthy pregnant women.

## Patients and Methods

### Patients

This study was conducted at Nanjing Maternal and Child Care Center, China. Healthy, pregnant women from our outpatient clinic at 20 to 36 weeks of gestation, and aged between 21 and 38, were enrolled from March 2009 to December 2010. A total of 724 patients were recruited. To minimize confounding factors, enrollment was dependent upon strict inclusion criteria: (1) primigravida with a healthy singleton pregnancy; (2) normal pre-pregnancy body mass index (BMI) ranging from 18.5 to 24.9 kg/m^2^; (3) confirmed gestational age (GA) of fetus, calculated according to a definite last menstrual period and then confirmed by routine ultrasonography at an early gestational age; (4) no known maternal obstetric or other medical problems, such as chromosomal abnormalities, autoimmune diseases, chronic or pregnancy-induced hypertension or diabetes mellitus.

### Ethics statement

This study was approved by the Medical Ethics Committee of Nanjing Maternal and Child Health Hospital. Women attending our hospital for conventional ultrasound examinations would read information about prenatal ultrasound examinations and the purpose of the study, and written informed consent was obtained from each participant for the examination. In the ultrasound examination, values for the BPD, HC, AC, FL and HUM parameters were routinely recorded, and the target parameters of ASTT and SSTT were additionally taken for a few seconds after verbal consent from all participants.

### Sonography

**Figure 1:**
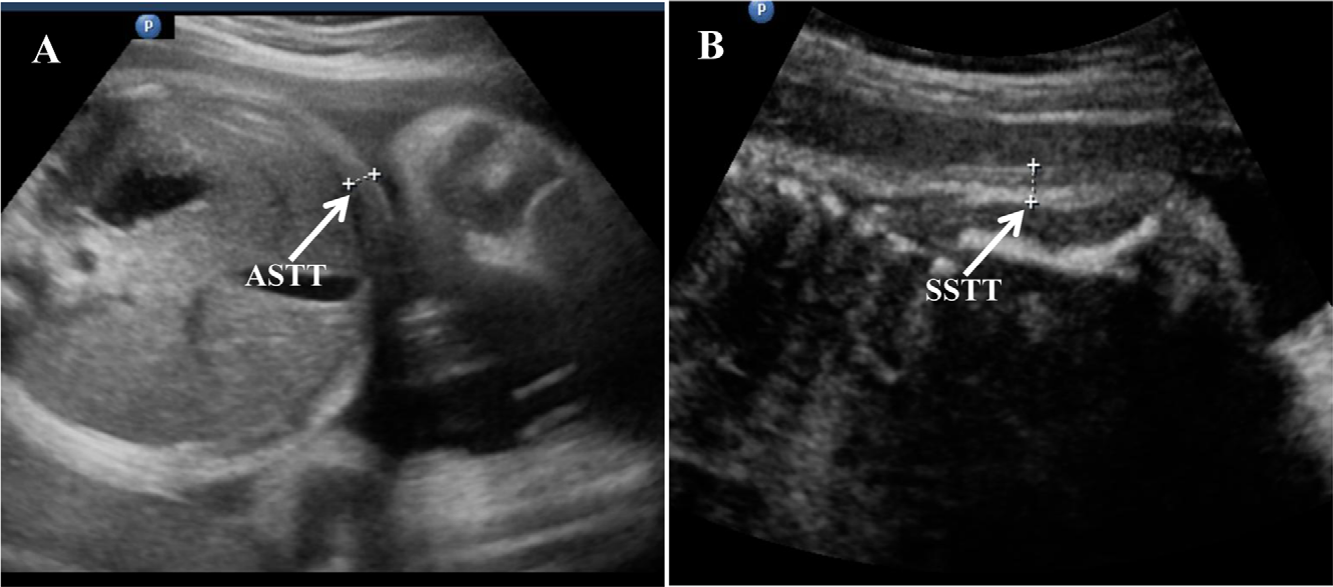
Ultrasound images of ASTT and SSTT. Images with arrows showing the measurement of (A) fetal abdominal subcutaneous tissue thickness (ASTT) and (B) fetal subscapular subcutaneous tissue thickness (SSTT).

All women recruited to the study underwent a conventional ultrasound examination using the commercially available Philip IU-22 Ultrasound Machine with a 3.15-MHz probe. During the evaluation, two qualified examiners recorded routine fetal ultrasonographic biometric parameters, including biparietal diameter (BPD), head circumference (HC), abdominal circumference (AC), femur length (FL), and humerus length (HUM), at each selected GA. Two fetal fat mass indices, ASTT and SSTT, were assessed using the technique of Rigano et al [11]. Briefly, ASTT was evaluated by measuring the thickness of the anterior abdominal subcutaneous tissue on the same axial image as that used for abdominal circumference measurement (Figure 1A). To measure SSTT, the fetus was imaged in a naturally prone or lateral posture, as far as possible, so the entire scapula was seen. The caliper was positioned between the skin surface and the subcutaneous tissue at the interface, perpendicular to the lowest end of the scapula, as shown in Figure 1B. All measurements were performed by two trained observers. To test the intra- and inter-observer reproducibility, the ASTT and SSTT parameters were assessed in 25 different images. The coefficients of variation for each parameter were calculated as 7.5% and 8.9% for ASTT and 8.4% and 9.1% for SSTT, respectively.

### Statistical analyses

Statistical analyses were performed using SPSS, version 19.0. All parameters, at each gestational age, were checked for normality using the Kolmogorov-Smirnov test, expressed as means ± SD, and the 5^th^, 50^th^ and 95^th^ percentiles of each parameter were calculated. The coefficients of correlation between ASTT and GA, and SSTT and GA were investigated using Pearson's linear regression analysis.

## Results

### Patients

In total, 724 patients were recruited; 22 patients were excluded from the study, as detailed below. The final sample sizes for each GA ranged from n = 13 to n = 148 are detailed in Table 1 and Table 2.

**Table 1:** Characteristics of BPD, HC and AC with increasing gestational age.

		BPD (cm)					HC (cm)					AC (cm)				
		Percentile					Percentile					Percentile				
GA (wk)	n	5th	50th	95th	mean	SD	5th	50th	95th	mean	SD	5th	50th	95th	mean	SD
21	25	4.90	5.12	5.47	5.15	0.18	17.62	18.72	19.37	18.69	0.56	15.62	16.35	17.23	16.46	0.62
22	55	5.27	5.45	5.71	5.46	0.18	19.38	20.00	20.70	19.95	0.81	16.51	17.53	18.61	17.57	0.63
23	67	5.54	5.77	6.00	5.77	0.14	20.18	21.16	21.98	21.20	0.56	17.82	18.62	19.60	18.66	0.54
24	117	5.78	6.06	6.42	6.06	0.19	21.58	22.10	23.04	22.19	0.46	18.76	19.76	20.76	19.75	0.64
25	148	6.08	6.38	6.64	6.38	0.17	22.54	23.17	23.89	23.21	0.44	19.41	20.74	21.80	20.66	0.72
26	98	6.47	6.70	6.97	6.70	0.15	23.50	24.19	25.05	24.24	0.47	20.48	21.59	22.55	21.59	0.65
27	37	6.70	6.94	7.09	6.90	0.15	24.34	25.02	25.51	25.03	0.37	21.49	22.52	23.77	22.54	0.80
28	20	7.11	7.31	7.56	7.32	0.16	25.72	26.14	27.15	26.35	0.56	22.29	23.43	24.62	23.43	0.74
31	13	7.60	7.93	8.08	7.87	0.17	28.28	29.10	29.38	28.90	0.41	26.66	27.40	28.26	27.45	0.58
32	54	8.01	8.25	8.53	8.27	0.17	28.84	29.65	30.74	29.67	0.54	27.02	27.94	29.12	28.06	0.71
33	25	8.18	8.46	8.68	8.43	0.17	29.90	30.40	31.86	30.53	0.62	28.20	29.70	31.58	29.77	1.17
36	43	8.82	9.14	9.57	9.18	0.22	31.75	32.26	33.22	32.32	0.43	31.31	32.33	34.01	32.35	0.85

Notes: GA, gestational age; BPD, biparietal diameter; HC, head circumference; AC, abdominal circumference.

**Table 2:** Characteristics of FL and HUM with increasing gestational age.

		FL (cm)					HUM (cm)				
		Percentile					Percentile				
GA (wk)	n	5th	50th	95th	mean	SD	5th	50th	95th	mean	SD
21	25	3.33	3.62	3.72	3.57	0.14	3.11	3.43	3.63	3.40	0.17
22	55	3.52	3.78	4.08	3.78	0.17	3.33	3.59	3.86	3.58	0.17
23	67	3.85	4.07	4.37	4.07	0.16	3.57	3.81	4.02	3.80	0.14
24	117	4.11	4.29	4.61	4.31	0.15	3.77	4.01	4.28	4.01	0.15
25	148	4.24	4.50	4.75	4.50	0.16	3.92	4.16	4.42	4.16	0.16
26	98	4.47	4.72	5.01	4.74	0.16	4.11	4.35	4.58	4.36	0.26
27	37	4.77	4.93	5.17	4.96	0.14	4.26	4.49	4.79	4.50	0.18
28	20	4.93	5.14	5.50	5.16	0.19	4.47	4.73	5.01	4.73	0.17
31	13	5.80	6.00	6.20	6.00	0.13	5.24	5.36	5.48	5.36	0.18
32	54	5.76	6.05	6.26	6.04	0.18	5.06	5.39	5.67	5.40	0.22
33	25	6.08	6.27	6.52	6.29	0.15	5.39	5.50	5.72	5.53	0.13
36	43	6.61	6.85	7.22	6.90	0.18	5.71	5.98	6.31	6.03	0.17

Notes: GA, gestational age; FL, femur length; HUM, humerus length.

### Sonography

To exclude potential macrosomia in normal body weight mothers, the conventional ultrasonographic parameters, BPD, HC, AC, FL and HUM, were measured for all fetuses. If any of these parameters lay outside the 5^th^–95^th^ percentile ranges for these parameters at each GA, according to the Chinese reference values for normal fetal development [16], the measurements taken from this fetus were excluded from the calculations. In total, 702, out of 724 samples, were used in the analysis. Table 1 and Table 2 give the measurements for each sonographic parameter at each GA.

**Figure 2:**
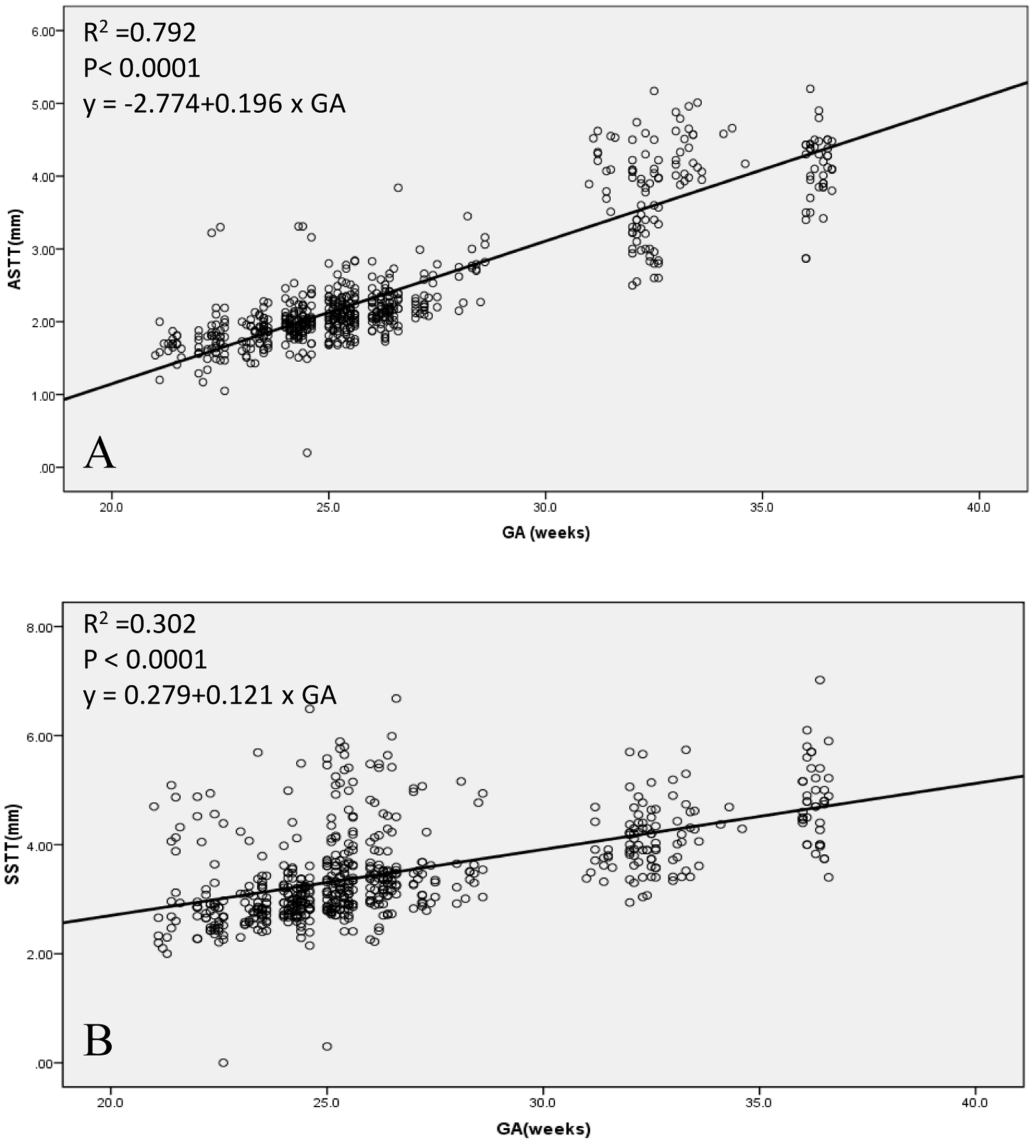
Relationships between GA and ASTT or SSTT. Linear regression relationships between gestational age (GA) and abdominal subcutaneous tissue thickness (ASTT; A), and GA and subscapular subcutaneous tissue thickness (SSTT; B).

Gestational ranges of fetal ASTT and SSTT were generated from fetuses of healthy pregnant mothers. Table 3 shows the 5^th^, 50^th^ and 95^th^ percentiles, as well as the means ± SD of these ranges. Both the ASTT and SSTT values increased progressively with advancing gestational age. Besides, a linear growth function was observed with increasing gestational age, and a first-degree regression correlation was found to exist between GA and ASTT (R^2^ = 0.792; P<0.0001; *y* = −2.774+0.196×GA; Figure 2A), and between GA and SSTT (R^2^ = 0.302; P<0.0001; *y* = 0.279+0.121×GA; Figure 2B).

**Table 3:** ASTT and SSTT measurements with increasing gestational age.

		ASTT (mm)					SSTT (mm)				
		Percentile					Percentile				
GA (wk)	n	5th	50th	95th	mean	SD	5th	50th	95th	mean	SD
21	25	1.43	1.69	1.86	1.66	0.17	2.12	3.32	4.84	3.42	0.97
22	55	1.33	1.79	2.19	1.78	0.37	2.28	2.85	4.90	3.10	0.86
23	67	1.52	1.87	2.18	1.84	0.20	2.42	2.84	4.95	3.11	0.76
24	117	1.66	1.98	2.40	2.01	0.33	2.56	2.99	4.97	3.19	0.75
25	148	1.72	2.10	2.55	2.13	0.25	2.71	3.35	5.68	3.69	1.01
26	98	1.86	2.18	2.63	2.22	0.28	2.71	3.46	5.89	3.84	1.02
27	37	2.10	2.34	2.92	2.41	0.27	2.86	3.68	6.17	4.15	1.18
28	20	2.22	2.71	3.17	2.66	0.34	3.01	3.59	7.02	4.31	1.34
31	13	3.62	4.21	4.58	4.16	0.36	3.36	3.76	4.53	3.80	0.39
32	54	2.60	3.40	4.53	3.53	0.62	3.22	4.02	5.09	4.08	0.58
33	25	3.93	4.33	4.94	4.37	0.35	3.41	4.28	5.28	4.24	0.64
36	43	3.40	4.18	4.77	4.12	0.47	3.74	4.79	5.90	4.80	0.74

Notes: GA, gestational age; ASTT, abdominal subcutaneous tissue thickness; SSTT, subscapular subcutaneous tissue thickness.

## Discussion

The intention of this study was to provide gestational growth trends and reference values for two fetal subcutaneous tissue thickness parameters, ASTT and SSTT, to aid in the assessment of fetal fat mass to evaluate fetal development and provide information on maternal gestational diabetes.

As previous studies have shown, several maternal factors, such as age, race, pre-pregnancy BMI, gestational weight gain, hypertension and diabetes, can influence the pattern of fetal body fat composition [17], [18]. Therefore, this study had strict inclusion criteria to ensure that these maternal factors did not influence the results. As we all know, maternal obesity is associated with a higher risk of macrosomia, compared to the risk in women with normal pre-gestational BMIs [19], [20]. However, even normal weight mothers can give birth to macrosomic babies; in a large population study carried out in the UK, the proportion of babies with birth weight above the 90^th^ centile was 9.03% [21]. To avoid macrosomia, in this study, fetal ASTT and SSTT measurements were included only for fetuses whose BPD, HC, AC, FL and HUM measurements were within the 5^th^–95^th^ percentile reference value ranges, and whose mothers had a normal pre-pregnancy BMI.

In neonates, fat mass accounts for about 46% of birth weight variance, despite the fact that fat mass constitutes only 12–14% of total body composition [22], which indicates the importance of the assessment of fetal fat mass. Bernstein et al. [23] found that fetal fat mass increases approximately ten-fold between 19 and 40 weeks of gestation. Their analysis of subcutaneous fat and lean body mass measurements in healthy fetuses, across the second and third trimesters, showed significant correlations with both birth weight and the neonatal lean and fat body mass ratio. Consequently, we examined two subcutaneous fat tissue parameters, SSTT and ASTT, in the last two trimesters in a population of healthy women with normal pregnancies.

Gestational diabetes mellitus (GDM) with carbohydrate intolerance is the most common metabolic disease of pregnancy [24]. It is well established that pregnancies with GDM are associated with increased obstetric complications, such as fetal macrosomia, neonatal hypoglycemia and hypocalcemia, as well as maternal hypertension and thromboembolic disease [25], [26]. Therefore, the surveillance of GDM during pregnancy is especially important.

As reported, ultrasound measurements of subcutaneous adipose tissue may be a reliable indicator of the fetal metabolic state in pregnancies with GDM [11], [27]. Meanwhile, in a comparison of SCTT values between fetuses from a population with GDM and those from a normal control group, there were significant differences between the initial fetal SCTT, but the difference disappeared if samples were controlled for maternal glucose levels during pregnancy [10], [11]. Moreover, Gojnic et al. found that fetal SCTT measurements appear to be a better predictor of maternal glucose control than the ambulatory glycemic profile [28]. Galan et al. [3] reported that reduced birth weight in normal fetuses born at a moderately high altitude (Denver, US), when compared with those born at sea level (Milan, Italy), was linked to a reduction in fetal subcutaneous fat tissue and abdominal fat tissue thickness, but not fetal lean mass. For these aspects, the availability of reference values for fetal subcutaneous fat ultrasound measurements may provide clinically useful information to identify excessive fetal fat deposition and evaluate maternal glucose levels in pregnancies with GDM.

In this study, the general trends of the ASTT and SSTT measurements with advancing gestational ages were parallel to those reported by Larciprete et al. [10]. However, the 50^th^ percentile ASTT measurement was smaller than that reported by Larciprete et al. and the 50^th^ percentile SSTT measurement was larger when adjusted for gestational weeks. We hypothesize that these differences were mainly due to the different ethnicities of the groups studied and the small sample size of our study. However, it is worth noting that some of our measurements did not correspond with the overall trend: for the ASTT measurements at 31 weeks, the 50^th^ percentile and the mean were higher than the values predicted by the leaner regression line; coupled with the fact that the 50^th^ percentile SSTT at 21 weeks was higher than expected and the mean SSTT measurements between 31 and 33 weeks were lower. These findings suggest that these standard reference values should be defined in a larger population. In addition, the thresholds of ASTT or SSTT values, which could distinguish between normal and abnormal fetuses, or be used, for example, to monitor maternal glycemic status, would need to be determined in well-designed large population studies of ethnically diverse populations, and involve individuals with both normal and abnormal pregnancies.

In conclusion, we performed an exploratory study to assess the general trends in fetal ASTT and SSTT measurements during the last two trimesters of pregnancy. These results provide preliminary ranges of normal ASTT and SSTT values as a reference to be used to evaluate fetal development and provide information on maternal metabolic factors, such as presence of uncontrolled gestational diabetes, in Chinese populations.
